# The prevalence and significance of substance use disorders in bipolar type I and II disorder

**DOI:** 10.1186/1747-597X-2-29

**Published:** 2007-10-01

**Authors:** Michael A Cerullo, Stephen M Strakowski

**Affiliations:** 1 The Division of Bipolar Disorders Research, Department of Psychiatry, University of Cincinnati College of Medicine, Cincinnati, Ohio, USA; 2 Department of Psychiatry, University of Cincinnati College of Medicine, Cincinnati, Ohio, 231 Albert Sabin Way (ML0559), Cincinnati, OH 45267-0559, USA

## Abstract

The aim of this paper is to provide a systematic review of the literature examining the epidemiology, outcome, and treatment of patients with bipolar disorder and co-occurring substance use disorders (SUDs). Articles for this review were initially selected via a comprehensive Medline search and further studies were obtained from the references in these articles. Given the lack of research in this field, all relevant studies except case reports were included.

Prior epidemiological research has consistently shown that substance use disorders (SUDs) are extremely common in bipolar I and II disorders. The lifetime prevalence of SUDs is at least 40% in bipolar I patients. Alcohol and cannabis are the substances most often abused, followed by cocaine and then opioids. Research has consistently shown that co-occurring SUDs are correlated with negative effects on illness outcome including more frequent and prolonged affective episodes, decreased compliance with treatment, a lower quality of life, and increased suicidal behavior. Recent research on the causal relationship between the two disorders suggests that a subgroup of bipolar patients may develop a relatively milder form of affective illness that is expressed only after extended exposure to alcohol abuse.

There has been very little treatment research specifically targeting this population. Three open label medication trials provide limited evidence that quetiapine, aripiprazole, and lamotrigine may be effective in treating affective and substance use symptoms in bipolar patients with cocaine dependence and that aripiprazole may also be helpful in patients with alcohol use disorders. The two placebo controlled trials to date suggest that valproate given as an adjunct to lithium in bipolar patients with co-occurring alcohol dependence improves both mood and alcohol use symptoms and that lithium treatment in bipolar adolescents improves mood and SUD symptoms.

Given the high rate of SUD co-occurrence, more research investigating treatments in this population is needed. Specifically, double blind placebo controlled trials are needed to establish the effectiveness of medications found to be efficacious in open label treatments. New research also needs to be conducted on medications found to treat either bipolar disorder or a SUD in isolation. In addition, it may be advisable to consider including patients with prior SUDs in clinical trials for new medications in bipolar disorder.

## Introduction

Bipolar I and II disorders are common and serious mental illnesses that have been reported to occur in approximately 1–3% [1,2] and 3–5% [3,4] of the US population respectively. Bipolar disorders are often complicated by co-occurring substance use disorders (SUDs). The aim of this paper is to provide a systematic review of the literature examining the epidemiology, outcome, and treatment of patients with bipolar disorder and co-occurring substance use disorders (SUDs). Articles for this review were initially selected via a comprehensive Medline search and further studies were obtained from the references in these articles. Given the lack of research in this field, all relevant studies except case reports were included.

The most thorough research in this field comes from epidemiological studies. Several large and well designed studies have consistently found high rates of co-occurring SUDs in bipolar disorder [2,5-9]. Several smaller studies have also added epidemiological information in specific populations [10-12]. Research in the last 2 decades has also focused on examining the effects of SUDs on the illness course of bipolar disorder [13-24]. Some recent studies also examined the sequence of onset of the two conditions to address questions of causality [14,18,19]. Finally, only a small number of studies have examined specific treatments in bipolar patients with SUDs [25-34].

### Epidemiology

Several studies investigated the prevalence of co-occurring SUDs in bipolar I and II disorders [5-10,35]. The largest study was conducted as part of the National Epidemiologic Survey on Alcohol and Related Conditions (NESARC) [2]. The NESARC examined the prevalence and co-occurrence of DSM-IV substance, mood, and anxiety disorders in a representative sample of 43,093 respondents in the US. In subjects with bipolar I disorder, there was a 58% lifetime prevalence of co-occurring alcohol use disorders and a 38% lifetime prevalence of any drug use disorder. Another large survey was conducted as part of the National Institutes of Mental Health Epidemiologic Catchment Area Program [5]. The prevalence of mental illness and SUDs was obtained from 20,291 persons from community and institutional settings. Among patients with bipolar type I disorder, 61% had a lifetime history of any drug or alcohol use disorder (46% had a lifetime history of an alcohol use disorder and 41% had a lifetime history of drug abuse or dependence) [5]. In patients with bipolar Type II disorder, 48% had a lifetime history of any drug or alcohol use disorder (39% had a lifetime history of an alcohol use disorder and 21% had a lifetime history of drug abuse or dependence). Among psychiatric illnesses, only anti-social personality disorder had a higher lifetime prevalence rate of any SUD (17% for the general population, 47% for schizophrenia, 27.2% for major depressive disorder, and 84% for anti-social personality disorder).

In another large study, Cassidy et al. [6] surveyed 392 patients who meet DSM-IIIR criteria for bipolar disorder in a mixed or manic episode. They found a lifetime prevalence of 48.5% for alcohol abuse, 24.2% for cocaine abuse, 4.6% for opioid abuse, and 36% for cannabis abuse. The point prevalence of SUDs at the time of the interview was 28.5% for alcohol abuse, 10.2% for cocaine abuse, 1.0% for opioid abuse, and 22.2% for cannabis abuse. McElroy et al. [7] evaluated 239 bipolar I and 49 bipolar II outpatients for any Axis I comorbidity. In bipolar I patients, the lifetime prevalence was 36, 10, 40, and 8 % for alcohol, cocaine, cannabis, and opioid use disorders respectively. In bipolar II patients, the lifetime prevalence was 22, 4, 10, and 0 % for alcohol, cocaine, cannabis, and opioid use disorders respectively. Chengappa et al. [35] interviewed 71 bipolar I and 18 bipolar II patients from a voluntary case registry of bipolar disorder. For bipolar I patients, the lifetime prevalence was 57.8, 11.3, 19.7, and 5.6 % for alcohol, cocaine, cannabis, and opioid use disorders respectively. For bipolar II patients, the lifetime prevalence was 38.9, 5.6, 5.6, and 0 % for alcohol, cocaine, cannabis, and opioid use disorders respectively.

Data regarding co-occurring SUDs and bipolar disorder have also been obtained from the two major first episode mania studies [8,9]. In The University of Cincinnati First-Episode Mania Study [8,14,15,36,37], the authors followed over a hundred patients who initially presented with a first manic or mixed episode and met DSM-IV criteria for bipolar type I disorder. The lifetime prevalence of alcohol and cannabis use disorders was 42% and 46%, respectively, in the 144 bipolar I patients studied [14,15]. Patel et al. [37] also used these data to examine differences in 103 early onset (age 12 to 17) and 58 typical onset (age 20 to 30) bipolar type I patients. Patients in the typical onset group were more likely to have a SUD (41%) compared to patients in the early onset group (24%). However, this finding may have been partly explained by the higher percentage of mixed episode presentations in the early onset group (67%) compared to the typical onset group (29%), because mixed patients were significantly less likely to have any co-occurring SUD.

The McLean-Harvard First-Episode Mania Study followed over a hundred patients for 2–4 years who initially presented with a first manic or mixed episode and met DSM-IV criteria for bipolar type I disorder [13]. Baethge et al. [9] used data from the first 112 patients from this study to examine co-morbid SUDs. All patients met DSM-IV criteria for bipolar type I disorder and presented to McLean hospital with a manic or mixed episode. The patients were followed for up to two years. At the index manic episode, thirty-seven (33%) of the patients were found to have a SUD with 22 patients using only one substance. The most common SUDs were alcohol abuse and dependence, followed by cannabis and then cocaine use disorders. Anxiety disorders were also found to be more frequent in patients with a SUD (30% vs. 13%). At the end of the 24 month follow up period, 31 out of the 80 remaining patients (39%) had a current SUD.

Two recent studies examined co-morbid SUDs and bipolar disorder in the Veterans Affairs (VA) hospital population [10,11]. Both studies used patient data from the Veterans Affairs (VA) Cooperative Study #450 (Reducing the Efficacy-Effectiveness Gap in Bipolar Disorder) [38,39]. Bauer et al. [10] examined 328 inpatients from 11 VA hospitals with bipolar I or II disorder from this study. Bipolar I and II patients were not analyzed separately but were grouped together for all prevalence calculations. They found a 33.8% current and 72.3% lifetime prevalence of a SUD. Alcohol use disorders had the highest lifetime prevalence (61.9%), followed by cannabis use disorders (22.6%), cocaine use disorders (19.5%), and then opioid use disorders (8.5%). Alcohol use disorders also had the highest point prevalence rates (25.6%), followed by cocaine use disorders (6.7%), cannabis use disorders (6.1%), and opioid use disorders (2.7%). Kilbourne et al. [11] used the VA study data to examine differences in bipolar type I disorder among minority populations. They examined 330 inpatients: 249 (76%) Caucasian; 47 (14%) African American; 26 (5%) Hispanic; 12 (4%) Native American; and 5 (1%) Asian/Pacific-Islander or other. Among the patients, 286 were diagnosed with bipolar type I disorder, 40 with bipolar type II disorder, and 1 with bipolar disorder NOS, and the three patient groups were combined in the analysis. Minority bipolar patients had a significantly higher number of cocaine use disorders (OR 2.2) and current alcohol abuse disorders (OR 1.8) compared to Caucasian patients. Among the minority groups, the only difference found was that cocaine use was significantly higher in African American patients (29%) compared with all other minorities (6%).

One recent study provided prevalence data for co-occurring SUDs in rapid-cycling bipolar patients. In this prospective study, Kupka et al. [12] followed 410 bipolar I, 104 bipolar II, and 16 bipolar disorder NOS patients over one year. Among these patients, 206 met the DSM-IV criteria for the rapid-cycling specifier. Rapid-cycling patients were significantly more likely to have a past diagnosis of drug (but not alcohol) abuse (27.3%) than non rapid-cycling patients (17.2%).

### Outcome

Recent research has increasingly focused on examining the effects that co-occurring SUDs have on illness course in bipolar disorder [13-24]. Many of the studies mentioned previously also addressed outcome questions, including the two major first episode mania studies [8,13]. Using data from the McLean Harvard First-Episode Mania Study [40,41], Tohen et al. [13] examined the clinical characteristics of 173 bipolar I patients who were followed for up to 5 years. They found that co-morbid SUDs were associated with increased episodes of depression. Strakowski et al. [8] and Strakowski et al. [36] examined the first 50 and 109 bipolar I patients respectively from The University of Cincinnati First-Episode Mania Study. Bipolar patients with co-morbid SUDs had increased treatment noncompliance compared to bipolar patients without any co-occurring SUD. Co-morbid SUDs had other direct negative effects even after adjusting for non-compliance (which was associated with poorer outcome), including a delayed onset of symptomatic recovery. In addition, alcohol abuse was positively correlated with the duration of depression, and the duration of cannabis abuse was positively correlated with the duration of mania.

Two more recent studies have used the University of Cincinnati data to examine the outcome of alcohol and cannabis use disorders on bipolar disorder [14,15]. These two studies attempted to investigate questions of causality (i.e. whether SUDs increase the risk of developing bipolar disorder or vice versa) by examining the sequence of onset of the two conditions. Although many patients had both alcohol and cannabis use disorders, each disorder had independent effects on the outcome of bipolar disorder [14,15]. Strakowski et al. [14] examined 27 bipolar I subjects in which the onset of an alcohol use disorder preceded the onset of bipolar disorder (Alcohol First) and 33 subjects in whom the bipolar disorder preceded or was concurrent with the onset of the alcohol use disorder (Bipolar First). The Bipolar First patients had more affective symptoms, earlier onset of bipolar disorder, and a slower recovery of affective symptoms compared to the Alcohol First patients. These results suggest that the Alcohol First patients may form a subgroup of bipolar patients that develop a relatively milder form of affective illness that is expressed only after extended exposure to alcohol abuse. Strakowski et al. [15] examined 33 bipolar I subjects in which the onset of a cannabis use disorder preceded the onset of bipolar disorder (Cannabis First) and 36 subjects in whom the bipolar disorder preceded or was concurrent with the onset of the alcohol use disorder (Bipolar First). The Cannabis First group did not differ from the Bipolar First group in terms of affective symptoms or age of onset of bipolar disorder. The Cannabis First group did show significantly higher rate of and more rapid recovery from affective episodes than the Bipolar First group. However, these differences did not remain after adjusting for potential mediator variables (including gender and age of onset, both of which were associated with time of recovery).

Three studies have used data from the Systematic Treatment Enhancement Program for Bipolar Disorder (STEP-BP) [42] to examine outcome in bipolar disorder with co-occurring SUDs [16-18]. Weiss et al. [16] examined the clinical characteristics of the first 1000 patients to enter STEP-BP, of which 105 had a current SUD, 332 had a past SUD, and 480 had no history of a SUD. Most (> 70%) patients met DSM-IV criteria for bipolar I disorder while the remainder of the patients were diagnosed with bipolar II, bipolar NOS, cyclothymia, or schizoaffective disorder. Compared with patients with no SUD, patients with a current or past SUD had a lower quality of life as measured by the Longitudinal Interval Follow-Up Evaluation-Range of Impaired Functioning Tool (LIFE-RIFT) and the quality of Life Enjoyment and Satisfaction Questionnaire. Patients with a current or past SUD also had a higher number of lifetime suicide attempts and were less likely to have a clinical status of recovering or recovered. Martinez et al. [17] analyzed the STEP-BP data and found that SUDs are significantly associated with all serious adverse events. Serious adverse events were defined as any event that "is fatal, life-threatening, disabling or incapacitating, requires or prolongs hospital stay, is a congenital anomaly in the offspring of a participant, or jeopardizes the participant or necessitates and intervention to prevent one of the aforementioned outcomes". Finally, Fossey et al. [18] used the first 1000 patients of the STEP-BP study to look at the validity of the distinction between primary and secondary SUD. A primary SUD precedes the onset of the bipolar disorder while a secondary SUD occurs after the onset of bipolar disorder. Of the 1000 patients, 116 had a primary SUD and 275 had a secondary SUD. They found that patients with a secondary SUD had fewer days of euthymia, more episodes of mania and depression, and a more suicide attempts than patients with a primary SUD. However, they found that these distinctions could all be explained by age at onset of bipolar disorder and therefore concluded that the primary secondary distinction is not valid. However, this study did not report how it defined age at onset, and moreover, many patients had been ill with both disorders for many years, making these results difficult to compare to first-episode studies. Winokur et al. [19] also examined the distinction between a primary and secondary SUD in bipolar disorder. They studied 30 bipolar patients (type unspecified) with primary alcohol use disorder and 34 bipolar patients with secondary alcohol use disorder. They found that the alcohol use disorder was significantly more likely to remit in the secondary SUD and patients with a primary SUD had significantly fewer episodes of affective episodes. Therefore they suggested that bipolar disorder associated with a primary SUD was a less severe form of illness that may require an alcohol use disorder to manifest.

In addition to the Weiss et al. study [16], three other outcome studies found that suicidal behavior is more likely in bipolar patients with SUDs than in those without [20-22]. Dalton et al. [20] examined 336 bipolar I and II and schizoaffective disorder (bipolar type) patients who were originally enrolled in a genetics study. They found that a history of a drug use disorder increased the risk twofold (OR = 2.1) for lifetime suicide attempts compared to patients without a history of SUD. They found no differences in rates of suicide attempts in patients with co-occurring alcohol use disorders compared to those without. In a retrospective study, Goldberg et al. [21] examined 100 patients diagnosed with dysphoric mania (patients were diagnosed with acute mania according to the DSM-IIIR and had ≥ 2 prominent concomitant depressive symptoms). Patients with a history of an alcohol use disorder were significantly more likely to report suicidal ideation. Swann et al. [22] examined 48 inpatients with bipolar disorder (type unspecified) in a cross-sectional study of impulsivity and suicidal behavior in bipolar disorder. They found that a history of alcohol abuse was associated with an increased probability of a suicide attempt. The authors suggested this behavior could be explained by increased impulsivity measures in patients with a history of alcohol abuse.

Two smaller prospective trials have examined outcome [23,24]. Tohen et al. [23] followed 75 patients diagnosed with bipolar disorder, manic type according to the DSM-III, for 4 years to investigate outcome after recovering from an episode of mania. A history of an alcohol use disorder was associated with an unfavorable outcome as defined by lower residential and occupational status. Singh et al. [24] examined 80 patients diagnosed with bipolar affective disorder using the International Classification of Diseases 10 (ICD-10). Half of the patients were also diagnosed with a co-occurring SUD. The bipolar patients with SUDs scored significantly lower on the World Health Organization Quality of Life (WHO-QOL) scale.

Finally, in a large chart review study, Feinman and Dunner [43] examined clinical characteristics and outcome measures in 188 bipolar I, II, NOS, and cyclothymic patients. One hundred and three patients had no history of SUDs (primary), 35 patients had a SUD which developed after the bipolar illness (complicated), and 50 patients had the onset of bipolar disorder after a SUD (secondary). The complicated group had the highest number of attempted suicides, and both the complicated and secondary group had significantly increased scores on the Hamilton Anxiety and Beck Depression rating scales.

### Treatment

Although bipolar disorder is complicated by SUDs in nearly half the total number of cases, there is a scarcity of research examining treatment in this specific population. In addition, most of these studies used open label designs and therefore should not be used to guide treatment [25-27,29,30,44,45]. Instead, successful open label studies should guide further research and be replicated using double-blind placebo-controlled studies. This problem is compounded because substance abuse is often an exclusionary criterion in many double-blind placebo-controlled bipolar treatments studies [22]. Given these limitations, Kosten and Kosten [31] reviewed medication strategies in bipolar patients with co-occurring SUDs. They suggested carbamazepine and valproate should be considered in these populations because both medications may have some benefit in patients with alcohol use disorders and valproate may also be effective in preventing cocaine relapse.

Two open label studies measuring responsiveness to lithium in bipolar patients found that co-occurring substance abuse was a predictor of a poor outcome [29,30]. O'Connell et al. [29] followed 248 patients diagnosed with bipolar disorder according to the DSM-III for at least one year. Co-occurring SUDs were associated with a lower Global Assessment Scale (GAS) score. Kusalic and Engelsmann [30] followed 29 patients diagnosed with bipolar disorder according to the DSM-III for up to two years. Co-occurring SUDs were a strong predictor of non-response. In contrast to O'Connell et al [29] and Kusalic and Engelsmann [30], Geller et al. [28] found that lithium may be useful in bipolar adolescents with a secondary SUD. They studied lithium versus placebo in 25 adolescents with bipolar disorder (12 with bipolar I, 5 with bipolar II, and 8 with major depressive disorder in the presence of future predictors for bipolar disorder) and a co-occurring SUD (dependence on drugs or alcohol). Patients in the lithium group had significantly greater improvement in both the affective symptoms and the SUD.

Salloum and colleagues have studied the effectiveness of valproate maintenance in bipolar type I patients with co-occurring alcohol [25] and cocaine dependence [46]. In the first study, a double-blind placebo-controlled trial, Salloum et al. [46] studied the effectiveness of treatment as usual (lithium) or lithium plus valproate in patients with co-occurring alcohol dependence. Subjects were followed for 24 weeks. Fifty nine patients who met DSM-IV criteria for current alcohol dependence and an acute episode of bipolar I disorder (either a manic, mixed or depressive episode) participated in the study. The HDRS and the Bech-Rafaelsen Mania Scale (BRMS) were used as outcome measures of affect while the number of heavy drinking days and the number of drinks per heavy drinking day were used to assess alcohol use. The valproate group had significantly lower numbers of heavy drinking days. The valproate group also had fewer drinks per heavy drinking day when medication adherence was added as a covariate. Both of these improved outcomes were correlated with higher valproate serum levels. There was no difference in the two groups on the HDRS or BRMS. The second study was an open label trial of divalproex in seven subjects with co-occurring cocaine dependence and an alcohol use disorder [25]. Subjects were started on divalproex and followed for 8 weeks. The authors found a significant increase in number of days abstinent from cocaine; a significant decrease in money spent on cocaine; a significant improvement in the Addition Severity Index drug severity index; and significant decreases in affective symptoms as measured by the HAMD and BRMS.

Brown and colleagues have studied multiple medications for use in bipolar patients with co-occurring SUDs [26,27,44,45]. Brown et al. [27] studied the effectiveness of aripiprazole treatment in an open-label study of bipolar type I patients suffering an acute mood episode with a co-occurring SUD. Twenty patients were enrolled in the study; 18 subjects met DSM-IV criteria for bipolar type I disorder; 1 met the criteria for bipolar II disorder; and 1 met the criteria for schizoaffective disorder, bipolar type. Eleven patients were experiencing a depressive episode, 6 a mixed episode, 4 a manic episode, and one patient did not meet criteria for a current mood state. All subjects had at least one co-occurring SUD which included alcohol dependence (N = 17), cannabis use disorders (N = 3), cocaine use disorders (N = 10), and opioid dependence (N = 3). All patients were switched from their previous treatment to aripiprazole and followed over 12 weeks. There was significant improvement in baseline-to-exit HDRS, YMRS, and BPRS scores at the end of the trial. In addition, patients with alcohol dependence had a significant decrease in dollars spent on alcohol and alcohol craving, and patients with cocaine dependence had a significant decrease in cocaine craving but not in cocaine use.

Brown et al. [45] examined the effectiveness of lamotrigine in an open label study with bipolar outpatients with co-occurring cocaine dependence. The study included 22 bipolar I, 7 bipolar II, and 1 bipolar not otherwise specified (NOS) subjects. Lamotrigine was titrated up to 300 mg/day and patients were followed for 12 weeks either as an add on or as a monotherapy if the patient was not taking any other medications. Ten patients were experiencing a depressive episode, 7 a mixed episode, 9 a manic episode, and three patients did not meet criteria for a current mood state. In addition to cocaine dependence, many subjects also had co-occurring alcohol and other substance use disorders. At the end of the study patients had significantly improved HDRS, YMRS, and BPRS. Cocaine cravings were significantly decreased as measured by the CCQ but there was no significant decrease in the dollar amount spent on cocaine.

Brown et al. [26] studied quetiapine add on therapy in a 12 week open label study of 14 bipolar type I and 3 bipolar type II patients with co-occurring cocaine dependence. At the end of the study there was a significant improvement in scores on the Hamilton Depressive Rating Scale (HDRS), Young Mania Rating Scale (YMRS), Brief Psychiatric Rating Scale (BPRS), and Cocaine Craving Questionnaire (CCQ). However, there was no significant change in dollars spent on cocaine, duration of cocaine use, and positive urine drug screens. Finally, Brown et al. [44] studied Naltrexone in an open label study in 34 bipolar outpatients with co-occurring alcohol dependence. Natltrexone was added as an adjunct to the patients existing psychotropic medications which included a variety of anti-depressants and mood stabilizers. The patients showed significant improvement in HAMD and YMRS scores and had significant decreases in alcohol use and craving.

Three studies have examined the effectiveness of psychotherapy as an adjunct to medications [33,34,47]. Drake et al. [47] examined the effectiveness of integrated dual disorder treatment in the state mental health system in bipolar patients with co-occurring SUDs. They followed 51 bipolar patients (type not specified) in the state mental health system for three years. Although there were few clinically significant improvements in psychiatric symptoms, there were significant clinical improvements in the substance abuse. Nearly two-thirds of the patients were in full remission from their SUD at 3 years. However, this study was limited by the lack of a comparison group. Schmitz et al. [33] randomly assigned 46 bipolar outpatients (type unspecified) to medication or medication plus cognitive behavioral therapy for 12 weeks. Patients in the therapy group reported significantly fewer days of manic or depressive symptoms. There were no differences in measures of drug and alcohol use or medication compliance. Weiss et al. [34] compared integrated group therapy versus group drug counseling in 62 bipolar patients (50 with bipolar I, 10 with bipolar II, and 2 with bipolar NOS). All patients had an alcohol or drug use disorder (dependence). Patients in the integrated group therapy treatment had significantly fewer days of substance use but had significantly more affective symptoms.

Finally, treatment is further complicated because the quality of care for SUDs is diminished in patients with bipolar disorder. Kilbourne et al. [48] examined 1,559 patients with mental illness and co-occurring SUD from the Nation Patient Care Database. The quality of the substance abuse treatment was assessed by measures of identification, initiation, and engagement of the SUD. Bipolar patients (23 percent of the total) were found to be less likely to initiate or continue treatment for a SUD compared to those with schizophrenia and schizoaffective disorder.

## Conclusion

Research has consistently shown that patients with bipolar I and II disorder have an extremely high rate of co-occurring SUDs (see Table 1). Among the bipolar spectrum, bipolar type I has consistently been shown to have higher rates of co-occurring SUDs than bipolar type II, bipolar NOS, and cyclothymic disorder [5-10,35]. Based on multiple large prior studies it can be conservatively estimated that bipolar I patients have at least a 40% lifetime prevalence of alcohol and other drug use disorders (with bipolar II patients having at least a 20% lifetime prevalence) [5-10,35]. The highest rate of SUDs was found in the VA population at 61.9 % lifetime prevalence of alcohol use disorders [10]. Alcohol and cannabis are the substances most often abused, followed by cocaine and then opioids [5-7], likely reflecting the general availability of these compounds, rather that diagnosis-specific selection.

**Table 1:** Epidemiology studies examining the prevalence of co-occurring SUDs in bipolar disorder.

Epidemiological Study	Number of subjects	Lifetime prevalence of co-occurring SUDs in bipolar I (or combined total if type not specified)	Lifetime prevalence of co-occurring SUDs in bipolar II
NESARC [2]	General Population; 43,093 subjects	58% for alcohol use disorders; 38% for drug use disorders	
National Institutes of Mental Health Epidemiologic Catchment Area Program [5]	General Population; 20,291 subjects	61% for any SUD	48% for any SUD
Cassidy et al. [6]	392 bipolar I patients	48.5% for alcohol abuse; 24.2% for cocaine abuse; 4.6% for opioid abuse; 36% for cannabis abuse.	
McElroy et al. [7]	239 bipolar I; 49 bipolar II	36% for alcohol abuse; 10% for cocaine abuse; 8% for opioid abuse; 40% for cannabis abuse.	22% for alcohol abuse; 4% for cocaine abuse; 0% for opioid abuse; 10% for cannabis abuse.
Chengappa et al. [35]	71 bipolar I; 18 bipolar II	57.8% for alcohol abuse; 11.3% for cocaine abuse; 5.6% for opioid abuse; 19.7% for cannabis abuse.	38.9% for alcohol abuse; 5.6% for cocaine abuse; 0% for opioid abuse; 5.6% for cannabis abuse.
The University of Cincinnati First-Episode Mania Study [14, 15]	144 bipolar I	42% for alcohol abuse; 46% for cannabis abuse	
The McLean-Harvard First-Episode Mania Study [9]	112 bipolar I	33% for any SUD during the index affective episode	
Veterans Affairs (VA) Cooperative Study #450 [10]	328 bipolar I and II patients (analyzed together)	61.9% for alcohol abuse; 19.5% for cocaine abuse; 8.5% for opioid abuse; 22.6% for cannabis abuse	

Research examining the effect of SUDs on illness course has been just as consistent and has clearly shown that co-occurring SUDs are associated with significant negative effects on outcome [8,13,16]. Patients with co-occurring SUDs have more and prolonged affective episodes and are less compliant with treatment. Four separate studies have also linked co-occurring SUDs with increased suicidal behavior [16,20-22]. Finally, patients with co-occurring SUDs have a lower overall quality of life compared to bipolar patients without SUDs [16]. However, a major limitation of these studies is the inability to address the causal relationship between the poor outcome and the SUD. Co-occurring SUDs may by contributing to the negative outcome: Alternatively, patients with poor outcome may have a more severe form of the disorder that also places them at risk for SUDs.

An interesting new line of research is examining whether patients who develop bipolar disorder after prolonged substance use may represent a subtype of bipolar disorder [14,18,19]. Fossey et al. [18] provided evidence that the effects of SUDs on bipolar disorder can be explained in terms of an earlier age of onset. However, Strakowski et al. [14] showed effects of alcohol use independent of age at onset. Strakowski et al [14] and Winokur et al [19] also found evidence that bipolar disorder that presents after a SUD may form a subgroup of bipolar patients that develop a relatively milder form of affective illness that is expressed only after extended exposure to alcohol abuse.

The scarce research regarding effective treatment in this patient population is far from conclusive. Most of the treatment studies were limited by an open label design and small numbers (see Table 2). The situation is further complicated because many placebo controlled clinical trials of bipolar disorder exclude patients with co-occurring SUDs. The two placebo controlled trial support the addition of valproate to lithium in bipolar patients with co-occurring alcohol dependence [25] and lithium treatment in bipolar adolescents with co-occurring SUDs [28]. Open label trials have provided limited evidence that quetiapine [26], aripiprazole [27], and lamotrigine [45] may be effective in treating affective and substance use symptoms in bipolar patients with cocaine dependence and aripiprazole may also be effective in patients with alcohol use disorders.

**Table 2:** Treatment studies of bipolar disorder with co-occurring SUDs.

Treatment Study	Design	Treatment	Number of patients	SUD	Results
Geller et al. [28]	double-blind placebo-controlled	Lithium	12 bipolar I; 5 bipolar II	Alcohol or drug dependence	Improvement in affective and SUD symptoms
Salloum et al. [46]	double-blind placebo-controlled	Valproate (as adjunct to lithium)	59 bipolar I	Alcohol dependence	Improvement in SUD but not affective symptoms
Brown et al. [27]	Open-label	Aripiprazole	18 bipolar I; 1 bipolar II; 1 schizoaffective disorder, bipolar type	Alcohol or drug abuse or dependence	Improvement in affective and SUD symptoms
Brown et al. [45]	Open-label	Lamotrigine	22 bipolar I; 7 bipolar II; 1 bipolar NOS	Cocaine dependence	Improvement in affective and SUD symptoms
Brown et al. [26]	Open-label	Quetiapine	14 bipolar I; 3 bipolar II	Cocaine dependence	Improvement in affective symptoms but not SUD symptoms
Brown et al. [44]	Open-label	Naltrexone	34 bipolar (type?)	alcohol dependence	Improvement in affective and SUD symptoms
Drake et al. [47]	Open-label	Integrated dual disorder treatment	51 bipolar (type unspecified)	Alcohol or drug abuse or dependence	Improvement in SUD but not affective symptoms
Schmitz et al. [33]	Open-label	Cognitive therapy as an adjunct to medication	46 bipolar (type unspecified)	Alcohol or drug abuse or dependence	Improvement in affective symptoms but not SUD symptoms
Weiss et al. [34]	Open-label	Integrated group therapy	50 bipolar I; 10 bipolar II; 2 bipolar NOS	Alcohol or drug dependence	Improvement in SUD but not affective symptoms

The research reviewed highlights the importance of co-occurring SUDs in bipolar disorders. Nearly half of the bipolar patients a physician treats will also have a SUD. Outcome research has revealed that the SUD will have significant adverse effects on illness outcome. Yet there are still many open questions about outcome including whether SUDs actually cause or contribute to the onset of bipolar disorder. The answers to these questions may necessitate changes in the way both bipolar disorder and SUD are treated. For example, certain populations may be especially vulnerable to the onset of bipolar disorder with substance use. It would be important to educate and aggressively treat such at risk populations. Therefore research addressing the casual nature of the relationship between bipolar disorder and SUDs should be given high priority.

In addition to outcome studies, more research examining specific treatments in this population is needed and should be given a high priority. Specifically, double blind placebo controlled trials are needed to establish the effectiveness of medications found to be efficacious in open label treatments. Only with double blind studies can we be confident in the effectiveness of the potential treatments successful in open label trials. New research also needs to be conducted on medications found to treat either bipolar disorder or a SUD in isolation. Double-blind placebo-controlled studies are needed to determine if these treatments are efficacious in dual diagnoses patients. This also raises the question of whether patients with co-occurring SUDs should be excluded from new clinical trials of medication for bipolar disorder. There may be compelling reasons to exclude patients with ongoing SUDs, but it might be advisable to include patients with prior SUDs and thereby better generalize the results to the actual patient population.

The epidemiological evidence for the extremely high rate of co-occurring SUDs in patients with bipolar disorder along with the outcome data suggest that neither illness can be ignored. Therefore some level of integrated treatment is needed, even if this simply means treating both disorders separately using the best evidence possible for each disorder. While some research suggests that certain medications may be efficacious for both disorders, it is still an open empirical question as to how much more treatment can be integrated. Until more research is available specifically targeting this population, physicians should treat each illness separately using the most effective treatment available.

## Abbreviations

SUD Substance use disorder

NOS Not otherwise specified

## Competing interests

The author(s) declare that they have no competing interests.

## Authors' contributions

MC and SS conceived of the review and MC prepared a preliminary draft of the manuscript. Both authors revised drafts of the manuscript and approved the final manuscript.
